# Apigenin Inhibits the Growth of Hepatocellular Carcinoma Cells by Affecting the Expression of microRNA Transcriptome

**DOI:** 10.3389/fonc.2021.657665

**Published:** 2021-04-06

**Authors:** Shou-Mei Wang, Pei-Wei Yang, Xiao-Jun Feng, Yi-Wei Zhu, Feng-Jun Qiu, Xu-Dong Hu, Shu-Hui Zhang

**Affiliations:** ^1^ Department of Pathology, Yueyang Hospital of Integrated Traditional Chinese and Western Medicine, Shanghai University of Chinese Medicine, Shanghai, China; ^2^ Department of Biology, School of Basic Medical Sciences, Shanghai University of Traditional Chinese Medicine, Shanghai, China

**Keywords:** apigenin, hepatocellular carcinoma, microRNA, transcriptome sequencing, pathway

## Abstract

**Background:**

Apigenin, as a natural flavonoid, has low intrinsic toxicity and has potential pharmacological effects against hepatocellular carcinoma (HCC). However, the molecular mechanisms involving microRNAs (miRNAs) and their target genes regulated by apigenin in the treatment of HCC have not been addressed.

**Objective:**

In this study, the molecular mechanisms of apigenin involved in the prevention and treatment of HCC were explored *in vivo* and *in vitro* using miRNA transcriptomic sequencing to determine the basis for the clinical applications of apigenin in the treatment of HCC.

**Methods:**

The effects of apigenin on the proliferation, cell cycle progression, apoptosis, and invasion of human hepatoma cell line Huh7 and Hep3B were studied *in vitro*, and the effects on the tumorigenicity of Huh7 cells were assessed *in vivo*. Then, a differential expression analysis of miRNAs regulated by apigenin in Huh7 cells was performed using next-generation RNA sequencing and further validated by qRT-PCR. The potential genes targeted by the differentially expressed miRNAs were identified using a curated miRTarBase miRNA database and their molecular functions were predicted using Gene Ontology and KEGG signaling pathway analysis.

**Results:**

Compared with the control treatment group, apigenin significantly inhibited Huh7 cell proliferation, cell cycle, colony formation, and cell invasion in a concentration-dependent manner. Moreover, apigenin reduced tumor growth, promoted tumor cell necrosis, reduced the expression of Ki67, and increased the expression of Bax and Bcl-2 in the xenograft tumors of Huh7 cells. Bioinformatics analysis of the miRNA transcriptome showed that hsa-miR-24, hsa-miR-6769b-3p, hsa-miR-6836-3p, hsa-miR-199a-3p, hsa-miR-663a, hsa-miR-4739, hsa-miR-6892-3p, hsa-miR-7107-5p, hsa-miR-1273g-3p, hsa-miR-1343, and hsa-miR-6089 were the most significantly up-regulated miRNAs, and their key gene targets were MAPK1, PIK3CD, HRAS, CCND1, CDKN1A, E2F2, etc. The core regulatory pathways of the up-regulated miRNAs were associated with the hepatocellular carcinoma pathway. The down-regulated miRNAs were hsa-miR-181a-5p and hsa-miR-148a-3p, and the key target genes were MAPK1, HRAS, STAT3, FOS, BCL2, SMAD2, PPP3CA, IFNG, MET, and VAV2, with the core regulatory pathways identified as proteoglycans in cancer pathway.

**Conclusion:**

Apigenin can inhibit the growth of HCC cells, which may be mediated by up-regulation or down-regulation of miRNA molecules and their related target genes.

## Introduction

Hepatocellular carcinoma (HCC) is one of the main types of primary liver cancer. According to the latest statistics, more than 300,000 people die of HCC every year in China. It is the fifth largest cancer in the world and the second leading cause of cancer-related death (1, 2). Early clinical diagnosis and cure rate for HCC are low, and the rates of postoperative recurrence, metastasis, and mortality are extremely high. Even in developed countries, the relative survival rate of HCC in five years is only 7% (3). Due to the occult onset and high malignant degree of liver cancer, most patients with liver cancer are diagnosed at advanced stages, with treatment options mainly relying on chemotherapy and targeted therapy. Systemic chemotherapy with Western medicine, such as Adriamycin, Epirubicin, Fluorouracil, Cisplatin and Mitomycin, etc. has high general efficacy but also high toxicity, and the targeted therapeutic drugs are expensive. Therefore, a new and more affordable method is urgently needed to eradicate HCC.

Apigenin, a natural flavonoid, exists in *Scutellaria barbata*, *Lobelia chinensis*, *Oldenlandia diffusa*, Centipeda, Rhizoma Polygontum Cuspidatum, Veratrum Nigrum, Semen Plantaginis, Caulis Trachelospermi, and other Chinese herbal medicine. It has low intrinsic toxicity and has potential anti-oxidant, anti-inflammatory, anti-viral, and anti-cancer properties (4). Studies have demonstrated that apigenin can inhibit the proliferation of HCC cells, induce cell differentiation and apoptosis, inhibit cancer cell invasion and distant metastasis, inhibit angiogenesis, regulate immunity, enhance the sensitivity and reduce toxicity of chemotherapies (5–11). However, the molecular mechanism of apigenin in regulating the growth, invasion, and metastasis of HCC cells is still superficial and needs to be further investigated.

MiRNAs are highly conserved single-stranded non-coding RNAs that are involved in the regulation of various cellular activities, such as cell proliferation, apoptosis, differentiation, inflammation, migration, and invasion, and play important roles in tumorigenesis (12). It has been reported that apigenin has a good anti-HCC pharmacological effect, but the target miRNA molecules and related genes regulated by apigenin in the prevention and treatment of HCC are indeterminate. Therefore, in this study, the inhibitory effect of apigenin on human hepatoma cell line Huh7 and Hep3B was determined *in vitro* and *in vivo*, and the differential expression profile of miRNAs in Huh7 cells treated with apigenin was analyzed and screened using next-generation miRNA sequencing technology. Finally, we investigated the miRNAs and potential target genes of apigenin associated with the growth inhibition of HCC cells using ClueGo plug-in in Cytoscape-3.7.1 software, and miRTarBase database, and explored the molecular mechanism of apigenin in HCC.

## Materials and Methods

### Reagent and Cell Line

Apigenin and human HCC cell line (Huh7 and Hep3B) were used in this study. The cell origins and specifications were described previously (13) and are provided in Supplementary Materials.

### Cell Proliferation and Colony Formation Assays

Cell proliferation and colony formation assays were executed as previously described (13) and are provided in Supplementary Materials. Each experiment was repeated three times in duplicates.

### Cell Cycle and Apoptosis Analysis

Huh7 and Hep3B cells were collected for cell cycle or apoptosis analysis according to the manufacturer’s instructions, as described previously (14) and supplied in Supplementary Materials.

### Transwell Invasion Assay

Cell culture inserts (8 μm pore size; Corning-Costar, USA) and Matrigel invasion chambers (Corning-Costar, USA) were used according to the manufacturer’s instructions. The transwell invasion assay was performed as previously described (13).

### Tumorigenicity in Nude Mice

The assay of tumorigenicity in nude mice as previously described (15) and in Supplementary Materials. Male BALB/c nude mice (weighing 18 - 20 g) were purchased from Shanghai Lingchang Biotechnology Co. Ltd. (Certificate No. 20180003007216) and were maintained under specific pathogen-free conditions. All experiments were performed according to the guidelines of the Committee on Protection, Welfare and Ethics of Experimental Animals in Yueyang Hospital of Integrated Traditional Chinese and Western Medicine affiliated to Shanghai University of Traditional Chinese Medicine (no. YYLAC-2019-036-4-2).

### Immunohistochemistry and Tunel assay

Immunohistochemistry and Tunel assay were performed according to the manufacturer’s instructions and as previously described (16) and in Supplementary Materials. The primary antibodies were supplied in **Supplementary Table 1**. Tunel apoptosis assay kit (Catalog No. C1098) was purchased from Beyotime Institute of Biotechnology (Jiangsu, China).

### MicroRNA Transcriptome Expression Analysis

The method was provided in Supplementary Materials. The criteria for differentially expressed miRNAs and mRNAs were set at cutoff value of >2.0 fold change, and a threshold of RPM >10 for miRNA and FPKM >0.3 for mRNA based on the ratio between the treatment and the control. MiRNA experiments were performed by the Shanghai YunXu Bio-tech Company, Shanghai, China.

### RNA Isolation and Quantitative Real-Time PCR (qRT-PCR) Analysis

Quantitative real-time PCR (qRT-PCR) was performed according to the manufacturer’s instructions and as described previously (17). Primer sequences were shown in **Supplementary Table 2**. All samples were run in triplicate, and the relative miRNA and mRNA expression levels were calculated according to the 2^-△△Ct^ method (14).

### Bioinformatics Analysis for the Characterization of miRNA and Related Target Genes

SPSS 24.0 software was used for K-means clustering analysis. The standardized reads of differentially expressed miRNAs between apigenin and control groups were clustered by the Heatmap.2 function in R language. The total number of comparative reads was used to standardize the comparative splicing reads (junction reads) for each sample with log_2_ conversion. The differentially expressed miRNAs across the experimental groups were selected based on a log_2_ fold-change (log_2_ FC) > 2.0 and P-value < 0.001. The target genes of differentially expressed miRNAs were predicted based on CLUEGO plug-in in Cytoscape software (version 3.7.1) and miRTarBase database. Gene ontology (Go) annotations, including the terms “biological process”, “molecular function”, and “cellular component,” and Kyoto Encyclopedia of Genes and Genomes (KEGG) pathway enrichment analysis were then carried out for the identified target genes through by DAVID tools (https://david.ncifcrf.gov/) (18).

### Statistical Analysis

All experiments were performed in triplicate and the results are presented as mean ± standard deviation (SD). Statistical analysis between the groups was performed by Student’s t-test using SPSS 24.0 software (SPSS, Armonk, NY, USA). A value of *P* < 0.05 was considered statistically significant.

## Results

### Apigenin Inhibits Huh7 and Hep3B Cell Growth

**Figure 1:**
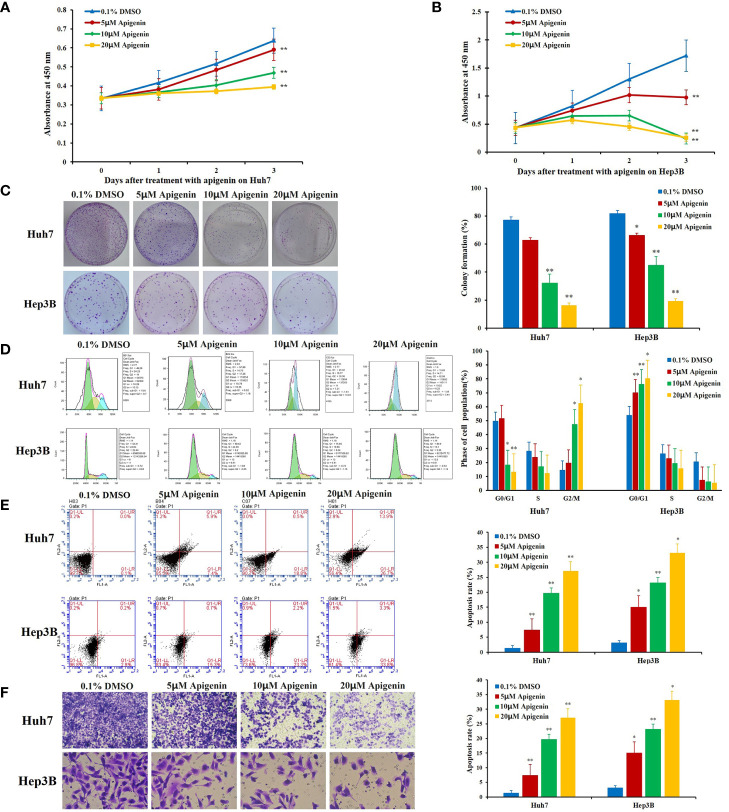
Effects of apigenin on the growth, colony formation, cell cycle progression, and apoptosis rate of Huh7 and Hep3B cells *in intro*. **(A**, **B)** Changes in cell viability as determined by the CCK-8 assay. **(C)** Colony formation. **(D)** Cell cycle distribution. **(E)** Apoptosis analysis by Annexin-V/PI staining. **(F)** Transwell invasion assay. ^*^ *P* < 0.05, ^**^ *P* < 0.01, indicating significant differences in comparison to the control treatment (0.1% DMSO) group. Data are shown as mean ± SD from three independent experiments.

To observe the effect of apigenin on the proliferation of hepatoma cells, we treated Huh7 and Hep3B cells with various concentrations (5, 10, and 20 µM) of apigenin for 3 days. We found that apigenin inhibited HCC cell proliferation in a dose-dependent manner (*P* < 0.05) (**Figures 1A, B** **)**. To investigate whether apigenin affected the ability of Huh7 and Hep3B cells to survive and form colonies, the same number of viable cells treated with apigenin or DMSO were seeded at a low cell density on the petri dish. After 14 days of culturing, the colonies were visualized and counted microscopically. As shown in **Figure 1C**, compared to the control group, apigenin significantly reduced the number and size of colonies formed in a concentration-dependent manner (*P* < 0.01).

### Apigenin Affects Cell Cycle Arrest and Apoptosis

**Figure 2:**
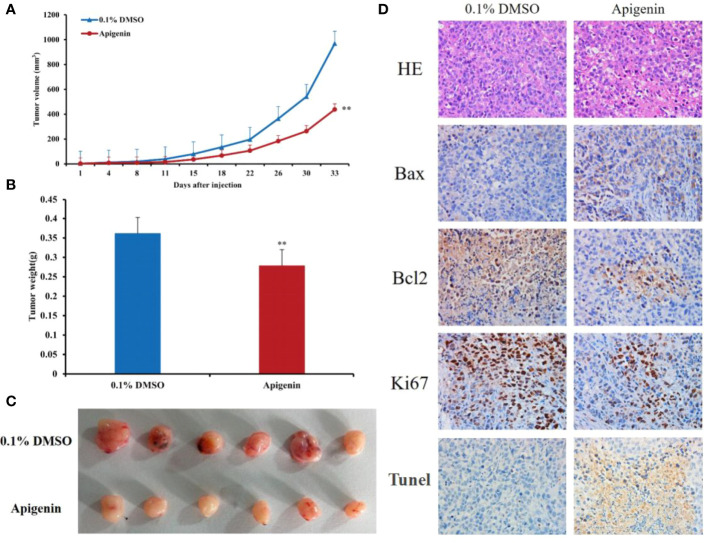
Effects of apigenin on the tumorigenicity of Huh7 xenograft in nude mice. **(A)** Tumor growth curves of Huh7 cells treated with apigenin (25 mg/kg/day) or vehicle control (0.1% DMSO). **(B)** Tumor masses of each treatment group. **(C)** Photographs of resected tumors (n = 6) from each treatment group. **(D)** Representative tumor sections stained with H&E, anti-Bax, anti-Bcl2, anti-Ki67 antibodies and Tunel assay kit. Original magnification: ×400. ^**^ *P* < 0.01 in comparison to the control treatment (0.1% DMSO) group.

To determine whether apigenin inhibits the growth of hepatoma cells by affecting cell cycle progression (G0/G1, S, and G2/M) and cellular apoptosis, we treated Huh7 and Hep3B cells with different concentrations of apigenin (5 µM, 10 µM, and 20 µM) for 48 h and investigated the cell cycle distribution by flow cytometry. The results showed that the number of G2/M phase cells increased and G0/G1 phase cells decreased significantly in Huh7 cells while the number of G0/G1 phase cells increased and G2/M phase cells decreased significantly in Hep3B cells (*P* < 0.05) (**Figure 1D**), suggesting that the inhibition of cell growth by apigenin is associated with cell cycle arrest. PI and annexin V staining was used to detect the effect of apigenin on cell apoptosis. The results displayed that the rate of apoptosis of Huh7 and Hep3B cells increased significantly after treatment with apigenin at different concentrations for 48 h (*P* < 0.01) (**Figure 1E**). This indicates that apigenin induces apoptosis in a dose-dependent manner. Then, apoptosis was detected by Tunel assay and the results displayed that the rate of apoptosis of tumor tissues from mice treated with apigenin increased significantly after treatment with apigenin (*P* < 0.01) (**Figure 2D**).

### Apigenin Restrains the Invasion of Huh7 and Hep3B Cells

Cell invasion assay provides a quantitative approach to study the invasion and metastasis of tumor cells induced by various cytokines. In this study, a transwell invasion experiment was performed to observe the effect of apigenin on the invasion ability of Huh7 and Hep3B cells. When observed under the microscope, the cells stained with crystal violet dye appeared rounded in shape. The invasion cells were more numerous in the control group but were sparse in the apigenin treatment group. Statistical analysis showed that compared with the control group, the invasion number of Huh7 and Hep3B cells treated with apigenin (5 µM, 10 µM, and 20 µM) was significantly reduced (*P* < 0.01) (**Figure 1F** and **Supplementary Table 3**). It is, therefore, suggested that apigenin is effective in inhibiting the invasion of Huh7 and Hep3B cells and is negatively correlated with the concentration of apigenin.

### Apigenin Suppresses Tumorigenicity of Huh7 Cells in Nude Mice

Next, we determined the effect of apigenin on tumorigenicity of Huh7 cells *in vivo*. In accordance with the findings *in vitro*, intraperitoneal injection of apigenin (25 mg/kg/day) suppressed tumor growth, showing a significant reduction in tumor volume and weight in comparison to the control treatment (0.1% DMSO) group (*P* < 0.05; **Figures 2A–C**). Microscopically, the tumor cells were arranged in diffuse compact trabeculae, with variable degrees of anaplasia and increased mitotic activity in the control group. In contrast, obvious necrosis was detected in the apigenin group. Immunohistochemical analysis of paraffin-embedded sections demonstrated increased Bax and decreased Bcl2 and Ki67 staining after apigenin treatment (**Figure 2D**).

### Apigenin Influenced miRNA Differential Expression as Assessed by Transcriptome Sequencing Analysis

**Figure 3:**
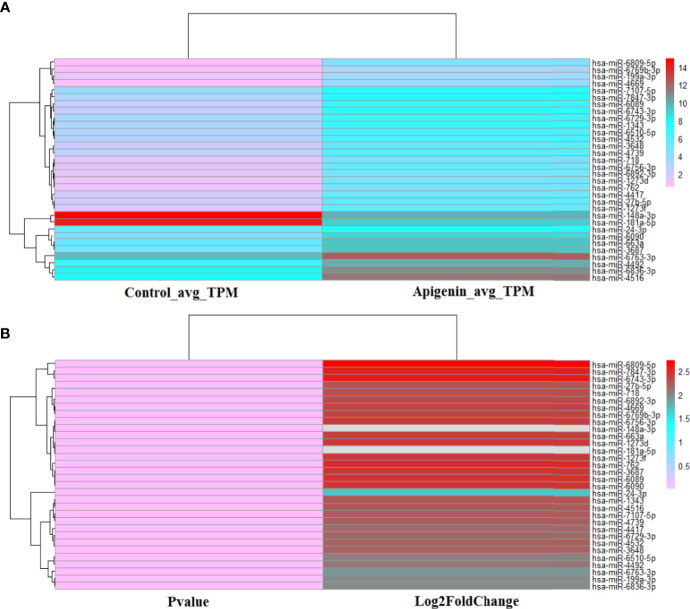
The heat map of 32 top apigenin-modulated miRNAs in Huh 7 cells. **(A)** The hierarchical clustering analysis was performed to analyze the average TPM expression of miRNAs in Huh 7 cells treated with 10 µM apigenin vs. 0.1% DMSO. **(B)** The hierarchical clustering analysis was performed to analyze the P value and Log2Fold of the 32 top apigenin-modulated miRNAs (P value ≤ 0.001^)^. Red indicates high relative expression and pink indicates low relative expression.

Under high-throughput RNA-sequencing (RNA-seq), the differentially expressed miRNAs in apigenin-treated Huh7 hepatocellular carcinoma cells were identified, of which 130 up-regulated and 9 down-regulated in apigenin treated cells (**Supplementary Table 4**). Further, we based on log_2_ fold-change (log_2_ FC) ≥ 2.0 and P value ≤ 0.001 selection criteria, a total of 32 miRNAs were obtained, of which 30 were up-regulated and 2 were down-regulated in apigenin treated cells (**Supplementary Tables 5**, **6**). The Heatmap.2 function of theR software package was used to cluster the differentially expressed miRNAs based on their expression profiles, and the results are shown as a hierarchical clustering heat map. As shown in **Supplementary Figure 1**, 139 differentially expressed miRNAs between the apigenin and control treatment groups could be effectively distinguished. We further made the heat map of 32 top apigenin-modulated miRNAs (**Figure 3**). Next, the miRNA target mRNA networks were constructed for the 30 upregulated and 2 down-regulated miRNAs using Cytoscape-3.7.1 software with the ClueGo plug-in and miRTarBase database (**Supplementary Figures S2** **,** **S3**).

### Confirmation of the Differentially Expressed miRNAs and Target Genes by qRT-PCR

**Figure 4:**
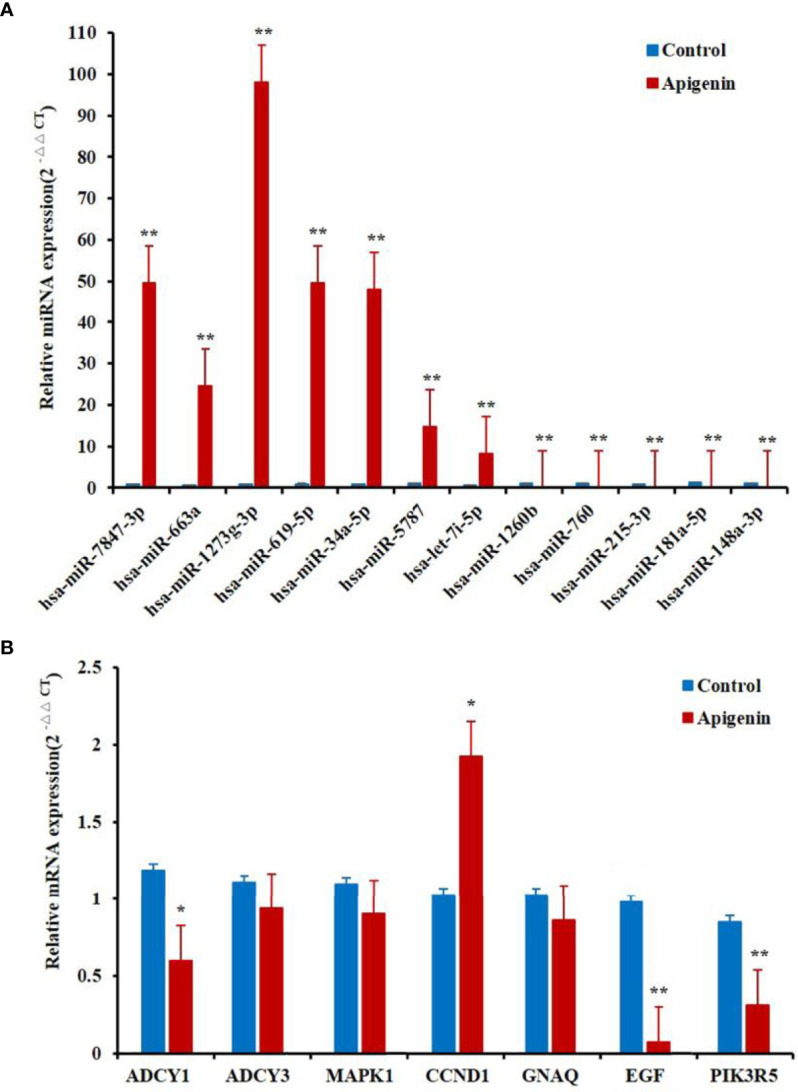
Differentially expressed miRNAs and related target genes between apigenin-treated cells and the control group by RT-PCR. **(A)** The verification of the RNA-seq data by qPCR analysis of 12 miRNAs. **(B)** The notarization of the RNA-seq data by qPCR analysis of seven genes. ^*^ *P* < 0.05 and ^**^ *P* < 0.01, compared with the control (0.1% DMSO) group. miRNAs and genes expression by RT-PCR.

To verify the RNA-seq data, qRT-PCR analysis was conducted for 12 selected differentially expressed miRNAs. The qRT-PCR and RNA-seq expression results were consistent, although there were no distinct variations statistically in the expression of some genes. The results of the differential expression analysis of miRNAs displayed that the expression of hsa-miR-7847-3p, hsa-miR-663a, hsa-miR-1273g-3p, hsa-miR-619-5p, hsa-miR-34a-5p, hsa-miR-5787, and hsa-let-7i-5p levels were increased in apigenin-treated cells as compared with the control cells. Conversely, hsa-miR-1260b, hsa-miR-760, hsa-miR-215-3p, hsa-miR-181a-5p, and hsa-miR-148a-3p expression was markedly down-regulated in the cells treated with apigenin (**Figure 4A**). In the differential gene expression level, only CCND1 gene were markedly up-regulated in apigenin-treated cells as compared with the control cells, the remaining 6 genes including MAPK1, PIK3R5, CCND1, ADCY1, ADCY3, GNAQ and EGF were down-regulated in the cells treated with apigenin as compared with the control group. However, there was no significant difference in MAPK1, ADCY3, and GNAQ gene (**Figure 4B**).

### Effect of Apigenin on Differentially Expressed miRNAs and Related Target Genes by KEGG Pathway Analysis

We further analyzed the enrichment of the KEGG pathways through the KEGG database. Then, we were able to infer the main pathways regulated by the differentially expressed miRNAs and their key target genes through KEGG pathway analysis.

**Figure 5:**
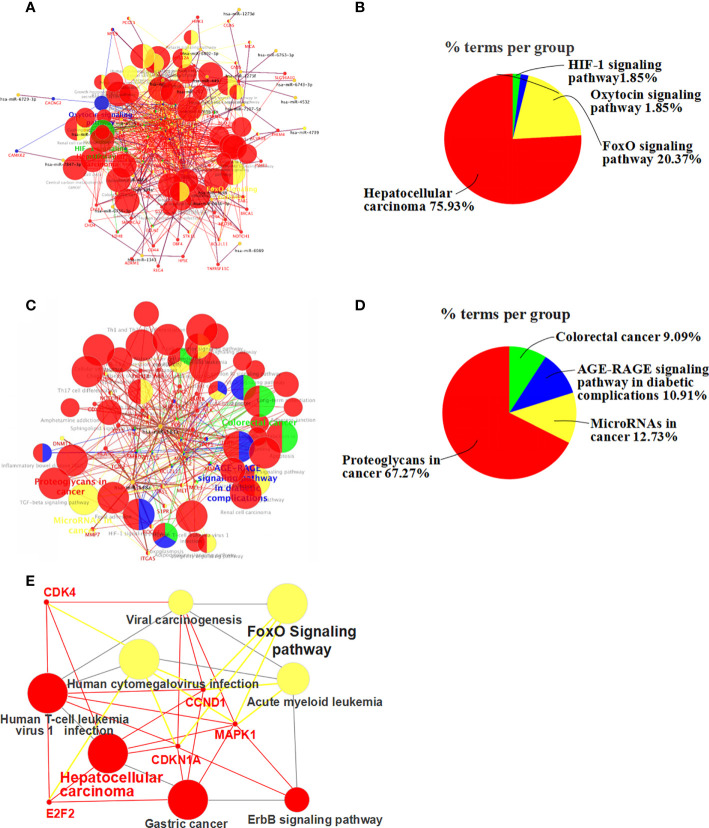
KEGG pathway enrichment analysis of differentially expressed miRNA-related target genes. **(A)** Up-regulated miRNA and associated target gene signaling pathways (up-regulated miRNAs-pathway-genes) in apigenin-treated Huh7 cells. **(B)** The percentage of the up-regulated miRNA-related signaling pathways. **(C)** Down-regulated miRNA and target gene signaling pathways in apigenin-treated Huh7 cells (down-regulated miRNAs-pathway-genes). **(D)** The percentage of the down-regulated miRNA-related signaling pathways. **(E)** Up-regulated miRNA-FoxO signaling pathways-genes in apigenin-treated Huh7 cells.

KEGG pathway enrichment showed that the effect of apigenin on the up-regulated miRNAs in Huh7 cells was associated with multiple signaling pathways and target genes (**Figure 5A**). These miRNAs were involved in a variety of cancer-related signaling pathways, such as hepatocellular carcinoma, pathways in cancer, cellular senescence, bladder cancer, proteoglycans in cancer, gastric cancer, melanoma, microRNAs in cancer, breast cancer, glioma, chronic myeloid leukemia, pancreatic cancer, FoxO signaling pathway, human T-cell leukemia virus 1 infection, human cytomegalovirus infection, colorectal cancer, endometrial cancer, small cell lung cancer, prostate cancer, PI3K-Akt signaling pathway, AGE-RAGE signaling pathway in diabetic complications, hepatitis B, non-small cell lung cancer, Kaposi sarcoma-associated herpesvirus infection, acute myeloid leukemia, Epstein-Barr virus infection, viral carcinogenesis, hepatitis C, human papillomavirus infection, and the erbB signaling pathway (**Supplementary Table 7**). The hepatocellular carcinoma pathway accounted for 75.93%, FoxO signaling pathway for 20.37% (**Figure 5B** **)**. Furthermore, MAPK1, E2F2, CDK4, CDKN1A, CCND1 were found key target genes of hepatocellular carcinoma pathway. MAPK1, CDKN1A, and CCND1 are key target genes of FoxO signaling pathway(**Figure 5E**). Meanwhile, apigenin up-regulated miRNAs and related target genes in Huh7 cells were arranged in descending order of Degree value. According to a Degree value > mean 9.7, the key target genes included MAPK1, PIK3CD, HRAS, CCND1, CDKN1A, E2F2, MYC, CDK4, MTOR, CDK2, STAT3, PTEN, CDKN1, BIGF1, MAPK14, TGFB1, CDKN2A, VEGFA, WNT4, CCNA2, MET, VAV3, APC2, and APC (**Supplementary Table 8**).

Similarly, the effect of apigenin on the down-regulated miRNAs in Huh7 cells was also related to a variety of signaling pathways and target genes (**Figure 5C**). These signaling pathways consisted of microRNAs in cancer, proteoglycans in cancer, cellular senescence, FoxO signaling pathway, PD-L1 expression and PD-1 checkpoint pathway in cancer, focal adhesion, Th17 cell differentiation, human T-cell leukemia virus 1 infection, natural killer cell mediated cytotoxicity, colorectal cancer, apoptosis, gastric cancer, AGE-RAGE signaling pathway in diabetic complications, T cell receptor signaling pathway, renal cell carcinoma, neurotrophin signaling pathway, Th1 and Th2 cell differentiation, Chagas disease (American trypanosomiasis), long-term potentiation, acute myeloid leukemia, yersinia infection, growth hormone synthesis, secretion and action, sphingolipid signaling pathway, Prolactin signaling pathway, melanoma, central carbon metabolism in cancer, Fc epsilon RI signaling pathway, non-small cell lung cancer, and VEGF signaling pathway (**Supplementary Table 9**). Proteoglycans in cancer accounted for 67.27%, microRNAs in cancer for 12.73% (**Figure 5D**). According to a Degree value > mean 7.2, the key target genes were MAPK1, HRAS, STAT3, FOS, BCL2, SMAD2, PPP3CA, IFNG, MET, and VAV2 (**Supplementary Table 8**).

### Effect of Apigenin on the Expression miRNAs and Related Target Genes by Go functional Analysis

**Figure 6:**
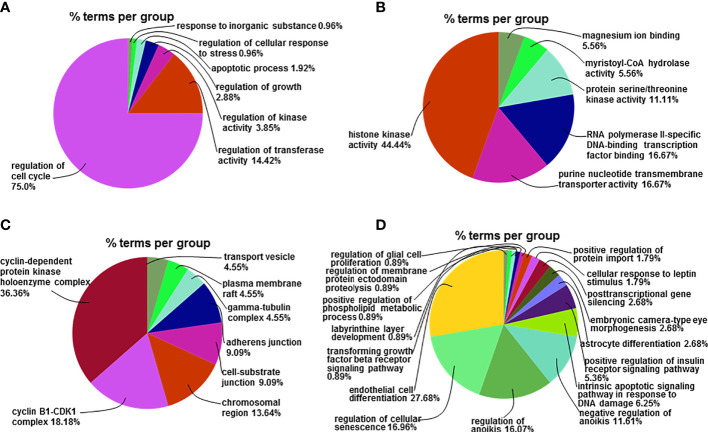
Go functional analysis of the differentially expressed miRNA-related target genes. **(A)** The percentage of the up-regulated mRNAs-related GO-BP terms enriched in the corresponding group in total up-regulated mRNAs-related GO-BP terms. **(B)** The percentage of the up-regulated mRNAs-related GO-MF terms enriched in the corresponding group in total up-regulated mRNAs-related GO-MF terms. **(C)** The percentage of the up-regulated mRNAs-related GO-CC terms enriched in the corresponding group in total up-regulated mRNAs-related GO-CC terms. **(D)** The percentage of the down-regulated mRNAs-related GO-BP terms enriched in the corresponding group in total down-regulated mRNAs-related GO-BP terms.

To understand the biological significance of the identified miRNAs and their target genes, functional analysis was performed using the Go database. Go enrichment analysis mainly included three parts: biological process (BP), molecular function (MF), and cell component (CC). We were able to annotate and infer the functions of the differentially expressed miRNAs by analyzing the Go functions, with a false discovery rate (FDR) ≤ 0.05 indicating biological significance. Compared with the control group, 88 Go-BP terms of miRNAs target genes up-regulated in the apigenin treatment group were mainly involved in Cell cycle regulation accounted for 75.0% of all biological functions, regulation of transferase activity for 14.42%, regulation of kinase activity for 3.85%, regulation of growth for 2.88%, apoptotic process for 1.92%, regulation of cellular response to stress for 0.96% and response to inorganic substance for 0.96% (**Figure 6A** **)**. Compared with the control group, 18 Go-MF terms of the miRNA target genes were up-regulated in the apigenin group and included histone kinase activity accounted for 44.44%, purine nucleotide transmembrane transporter activity for 16.67%, RNA polymerase II specific DNA-binding transcription factor binding for 16.67%, protein serine/threonine kinase activity for 11.11%, and myristoyl-CoA hydrolase activity and magnesium ion binding for 5.56% (**Figure 6B** **)**. Compared with the control group, 18 Go-CC terms of miRNA target genes up-regulated in the apigenin group were mainly involved in cyclin-dependent protein kinase holoenzyme complex accounted for 36.36%, cyclin B1-CDK1 complex for 18.18%, chromosome region for 13.64%, cell-substrate junction for 9.09%, adherens junction for 9.09%, gamma-tubulin complex, and plasma membrane raft and transport vesicle for 4.55% (**Figure 6C** **)**.

Compared with the control group, 82 Go-BP terms of miRNA target genes down-regulated in the apigenin group were primarily associated with endothelial cell differentiation accounted for 27.68% of all biological functions, regulation of cellular senescence for 16.96%, regulation of anoikis for 16.07%, negative regulation of anoikis for 11.61%, intrinsic apoptotic signaling pathway responding to DNA damage for 6.25%, and positive regulation of insulin receptor signaling pathway for 5.36% (**Figure 6D**). Only 2 Go-MF terms of the miRNA target genes were down-regulated in the apigenin group with phosphotyrosine residue binding accounted for 100% of the Go terms enriched. There were no Go-CC terms found for the down-regulated miRNA target genes in the apigenin group.

## Discussion

In this study, we demonstrated that apigenin inhibits the growth and invasion of HCC cells both *in vitro* and *in vivo*. *In vitro*, this effect showed that apigenin reduced HCC cell proliferation activity, induced cell cycle arrest and apoptosis, and suppressed the invasion of Huh7 and Hep3B cells. However, the effect of apigenin on cell cycle was completely different in Huh7 and Hep3B cells, suggesting that apigenin may have different effects on cell cycle regulation in different cell lines. Other studies have also confirmed that apigenin can induce the arrest of HepG2 cells in the G2/M phase and induce apoptosis (19).After the treatment of Bel-7402/adriamycin (ADM) cells, apigenin can induce cell arrest in the S phase (20). In addition, apigenin plays a role in other cancers by inducing apoptosis and cell cycle regulation (21, 22). *In vivo*, the inhibitory effect was manifested as apigenin halting the tumorigenicity of Huh7 cells in nude mice by decreasing the tumor volume, weight and increasing apoptosis, and impacting the expression of Bax, Bcl-2, and Ki-67. Therefore, apigenin has antitumor effects, which are consistent with the previous data demonstrated in various cancers, especially HCC (17).

Under high-throughput RNA-sequencing, we showed that apigenin-treated Huh7 cells were affected at the miRNA expression level. Apigenin up-regulated miRNAs were involved mainly in the hepatocellular carcinoma-related pathway and the FoxO signaling pathway, indicating that most of the differentially expressed miRNAs regulated by apigenin are related to the occurrence and development of HCC. Overall, the cell cycle regulation made up a large percentage of the Go terms. The differential expression of hsa-miR-199a-3p, hsa-miR-663a, and hsa-miR-24 in apigenin-treated Huh7 cells was mainly related to the proliferation and invasion of hepatoma cells. MiR-199a/b-5p was reported to inhibit the activation of the ROCK1/MLC and PI3K/Akt signaling pathways by negatively regulating ROCK1 expression, leading to the inhibition of liver cancer metastasis (23). MiR-663a/b can inhibit the proliferation and invasion of HCC cells by regulating TGF-β 1 and target gene GAB2 (24, 25). MiR-24 is known to promote HCC cell growth, metastasis, and invasion by targeting P53 or metallothionein 1M (26, 27), while our results demonstrated that apigenin up-regulated the expression of hsa-miR-24 and inhibited the growth of Huh7 hepatoma cells. In addition, up-regulation of miRNAs, such as hsa-miR-6769b-3p, hsa-miR-6836-3p, hsa-miR-4739, hsa-miR-6892-3p, hsa-miR-7107-5p, hsa-miR-1273g-3p, hsa-miR-1343, and hsa-miR-6089, by apigenin have not been reported previously.

The down-regulated miRNAs in apigenin-treated Huh7 cells were found to be involved mainly in proteoglycans in the cancer-related pathway and microRNAs in the cancer-related pathway. Of the identified Go terms, the differentiation of endothelial cells and apoptosis make up a large percentage. The down-regulation of hsa-miR-181a-5p and hsa-miR-148a-3p in apigenin-treated Huh7 cells was likely associated with the apoptosis and invasion of tumor cells. The deletion of the miR-181 family is known to inhibit the migration of tumor cells through the regulation of MAPK (28). In addition, miR-148b is found to suppress the proliferation, migration and invasion of HepG2 and SMMC 7721 cells by targeting Rho-associated protein kinase 1 (29). In this study, apigenin down-regulated the expression of hsa-miR-148a-3p and inhibited the proliferation of Huh7 hepatoma cells, which is inconsistent with the report in the literature. This discrepancy may be due to the different roles hsa-miR-148a-3p plays in different hepatoma cells. Although down-regulation of hsa-miR-148a-3p could promote cell proliferation and invasion, apigenin appears to affect multiple miRNAs at the same time, which results in the inhibition of the proliferation and invasion of hepatoma cells.

The miRNAs up-regulated in Huh7 cells during apigenin treatment were mainly linked to the suppression of MAPK1, PIK3CD, HRAS, CCND1, CDKN1A, E2F2, MYC, CDK4, MTOR, CDK2, STAT3, PTEN, CDKN1, BIGF1, MAPK14, TGFB1, CDKN2A, VEGFA, WNT4, CCNA2, MET, VAV3, APC2, and APC expression. BIGF1 is a newly discovered gene in HCC during this study. Among the down-regulated genes, CCND1 was found to be down-regulated by HOTAIR gene knockout Huh7 cells, which inhibited the proliferation and induced cell cycle arrest in Huh7 cells (30). Apigenin inhibited HCC growth by down-regulating CDK4 and up-regulating CyclinD1 *via* p38 MAPK-p21 signaling in Huh7, SMMC-7721 and HepG2 cell lines (31). Down-regulation of CDK2, cyclin A, cyclin B1 and cyclin E contributed to the anti-proliferation effect of apigenin treatment in human bladder cancer T-24 cells (32). In addition, MTOR, STAT3, HRAS and c-MYC as oncogenes, together with the transcription factor E2F2, play important roles in the proliferation, differentiation, apoptosis and invasion of HCC cells (9, 33, 34). However, in disagreement with our findings, an increase in CDKN1A expression was found to promote cell apoptosis (35). Overall, the predicted results of apigenin on miRNA target genes are consistent with the reported genes described in the literature. MYC, CDKN2A, PTEN, HARS, APC2, and APC are often referred to as miRNAs in cancer. CDKN2A and PTEN genes are involved in the p53 signaling pathway. WNT4, HGF and TGFB1 are associated with the molecular signaling pathways in cancer, proteoglycans in cancer, and the relaxin signaling pathway. CCND1, CDK4, MAPK1, CDKN1A, and E2F2 genes are linked to the development of hepatocellular carcinoma. At present, limited studies on PIK3CD, HRAS, E2F2, BIGF1, WNT4, and VAV3 genes have been reported in hepatoma cells, indicating the need for further research.

The down-regulated miRNAs in apigenin treated Huh7 cells showed anti-HCC pharmacological effects by increasing the expression of MAPK1, HRAS, STAT3, FOS, BCL2, SMAD2, PPP3CA, IFNG, MET, and VAV2. Of which, SMAD2, PPP3CA, MAPK1, and FOS genes are known to be involved in the regulation of tumor cell proliferation and invasion. Activation of TGF-β1/Smad2 signaling can promote epithelial-to-mesenchymal transition (EMT) and invasion of HCC (36). PPP3CA was down-regulated in the chip analysis of HCC patients with hepatitis C (37). The expression of MAPK1 inhibits the proliferation of Huh7 and Hep-G2 hepatoma cells (38). De-repression of c-Fos gene expression caused by miR-139 down-regulation contributes to MHCC97H cell metastasis (39). Inhibiting the expression of VEGF, VAV2, and CDC42 contributed to the suppression of angiogenesis and metastasis of HCC (40). PPP3CA is related to cell senescence, VEGF signaling pathway, etc. IFNG and VAV2 are associated with HIF-1 signaling pathway, TGF-β signaling pathway, etc. These genes have been rarely reported in liver cancer, which suggests the need for further study. In addition, we found that key target genes such as MAPK1 were included in the up-regulated and down-regulated miRNA networks, but our experimental results showed that there was no significant difference in MAPK1 after apigenin treatment, which may be caused by the mutual canceling of the up-regulated and down-regulated effects.

In summary, our study demonstrated that apigenin has an inhibitory effect in hepatoma cells, which is associated with anti-proliferation, induction of cell cycle arrest and apoptosis, and the suppression of HCC cell invasion. In addition, as a promising drug, apigenin has a variety of properties including obvious antioxidant and anti-inflammatory activity (41, 42). It is important to note that the effects of apigenin vary with different doses, and multiple studies have shown that 20-100 μM apigenin inhibits cell proliferation, invasion, and apoptosis induction (43, 44). It has also been suggested that high doses of apigenin may cause oxidative stress-induced liver damage (41). Therefore, in the application of the grasp of the dose is very important. This study is the first to utilize the whole genome expression profiles to identify apigenin-regulated miRNAs in HCC cells. These differentially expressed miRNAs may be involved in the specific molecular mechanism of the inhibitory activity of apigenin in hepatoma cells.

## Data Availability Statement

The authors acknowledge that the data presented in this study must be deposited and made publicly available in an acceptable repository, prior to publication. Frontiers cannot accept an article that does not adhere to our open data policies.

## Ethics Statement

The animal study was reviewed and approved by Yueyang Hospital of Integrated Traditional Chinese and Western Medicine, Shanghai University of Chinese Medicine, Shanghai, China.

## Author Contributions

X-DH and S-HZ designed the study. S-MW, P-WY, and X-JF contributed equally to this research as co-first authors. F-JQ and Y-WZ contributed to animal rearing. All authors contributed to the article and approved the submitted version.

## Funding

This study is supported by the National Natural Science Foundation of China (grant number: 82003994, 81172311); Highland Project from Integrated Traditional Chinese and Western medicine Branch of Shanghai University of Traditional Chinese Medicine; the special research project of traditional Chinese medicine in Henan Province (No. 2019JDZX2034).

## Conflict of Interest

The authors declare that the research was conducted in the absence of any commercial or financial relationships that could be construed as a potential conflict of interest.
