# Supplementary material for: Apigenin Inhibits the Growth of Hepatocellular Carcinoma Cells by Affecting the Expression of microRNA Transcriptome
Source: Front Oncol. 2021 Apr 6;11:657665. doi: 10.3389/fonc.2021.657665 (PMC8095173; doi:10.3389/fonc.2021.657665)
Supplement: Supplementary file 1 [file DataSheet_1.doc]

Supplementary Material

# Supplementary Data

**Supplemental Materials and methods**

## Cell lines

Apigenin was purchased from the China Food and Drug Administration (>95% of purity). Human HCC cell line (Huh7 and Hep3B) were purchased from the Institute of Cellular Research, Chinese Academy of Science, Shanghai, China. Cell were conventional cultured in DMEM (dulbecco’s modified eagle media) high sugar liquid medium (HyClone Co, USA) supplemented with 10% fetal bovine serum (FBS) (Gibco BRL, USA), 50U/mL penicillin, and 50mg/mL streptomycin at 37℃ in a 5%CO_2_ humidified incubator.

## Cell proliferation and colony formation assays

Huh7 and Hep3B cells (3×10^4^ cells/well) were seeded in 96-well plates and treated with apigenin (5 µM, 10 µM, and 20 µM) or 0.1% DMSO vehicle control. After incubation for 7 days, cell proliferation was measured using a 10% cell counting kit-8 (CCK-8, Signalway antibody Co, Beijing, China) solution prepared in serum-free essential medium. Briefly, 10 μl of the prepared CCK-8 solution was added to each well and incubated for another 1 h at 37°C. The absorbance was then measured at 450 nm using a microplate reader (Bio-Rad). IC50 (half maximal inhibitory concentration) values were calculated using SPSS (Statistical Package for the Social Sciences) IBM software (SPSS, Chicago, IL, USA). The colony formation assay was performed as previously described.

## Cell cycle and apoptosis analysis

Huh7 and Hep3B cells were seeded in 6-well plates at a density of 3×10^5^ cells per well and treated with apigenin (5 µM, 10 µM, and 20 µM) or 0.1% DMSO for 48 h. Flow cytometry analysis of DNA content and cellular apoptosis were examined using an Annexin V-FITC/Propidium Iodide Staining Kit (Biyuntian Biotechnology Co, Shanghai, China) according to the manufacturer's instructions. Stained cells were analyzed on an Accuri C6 Flow Cytometer (Becton Dickinson, San Jose, CA, USA) and data was analyzed with the FLOW JO software (FlowJo, Ashland, OR).

## Tumorigenicity in nude mice

Mice were randomly subdivided into 2 groups with 6 mice in each group. Huh7 cells (2×10^6^ cells/mouse) in 100 µL of serum-free culture medium were injected subcutaneously into the upper flank region of nude mice for tumor growth study. Tumor volumes were measured every 5 days with an electronic vernier caliper and calculated according to the following formula: 0.5 × length × width^2^. Apigenin (25 mg/kg/day) was delivered intraperitoneally for 20 days after 7 days following the cell inoculation. Orbital vein blood was then collected from the mice before the animals were sacrificed, and all tumors were excised for histological analysis. The tumors were fixed in 10% formalin, embedded in paraffin, sectioned, and subjected to standard hematoxylin and eosin (H&E) staining and processed for immunohistochemical staining.

## Immunohistochemistry and Tunel assay

The dehydrated paraffin slices (3 μm) were bleached and pasted in 45°C water bath, and then continuously baked for 20 minutes at 72°C. LeicaBond-Max automatic immunohistochemistry instrument (Beijing Haonos Technology Co., Ltd, Beijing, China) was used for staining, and the baked paraffin sections were automatically stained for Bax, Bcl-2 and Ki-67, using the corresponding reagents. The parameters used were as follows: heating antigen for 30 min, washing with PBS for 5 min after cooling, 3% H_2_O_2_ for 5 min, primary antibody incubation for 20 min, post primary for 10 min, polymer for 10 min, DAB staining for 10 min, and hematoxylin counterstain for 10 min. After the staining procedure, the tissue sections were dehydrated by gradient ethanol( 75%, 85%, 95%, 100%), sealed and photographed under the microscope. The staining results were blindly evaluated by two experienced pathologists without knowledge of the information of the sections.

Dewaxed sections were incubated with proteinase K at 37°C for 15–30 minutes to permeabilize them, then rinsed in PBS. Sections were placed in 3% H_2_O_2_ for 10 minutes at room temperature and washed in PBS. Following incubation with 50 μL labeling buffer per sample for 1 hour at 37°C in a humidified chamber, and a PBS wash, the labeling reaction was performed using 0.2 mL labeling reaction termination liquid per sample, and incubated for 10 minutes at room temperature followed by another PBS wash. Slices were incubated with streptavidin-horseradish peroxidase solution for 30 minutes at room temperature in a humidified chamber. After another PBS wash, the sections were placed in diaminobenzidine solution and washed again with PBS before being dehydrated in alcohol and mounted. The staining results were blindly evaluated by two experienced pathologists without knowledge of the information of the sections.

## MicroRNA transcriptome expression analysis

Total RNA from three independent Huh7 cell cultures treated with 10 µM apigenin or 0.1% DMSO (control) for 48 h was isolated using Trizol reagent (Invitrogen, Carlsbad, CA, USA) and the RNeasy kit (Qiagen) according to manufacturer’s instructions, including a DNase digestion step. RNA quantity and purity were determined to ensure an OD260/OD280 ratio was within the range of 1.8 - 2.0 and an rRNA ratio of 28S/18S > 1.0 using a ND-1000 spectrophotometer (NanoDrop Technologies, Wilmington, DE, USA) and denaturing gel electrophoresis, respectively. The samples were amplified and labeled using an Agilent 2100 Bioanalyzer before progressing through the library preparation. The library construction was prepared using a TruSeq Small RNA Sample Preparation Kit. The 3’ and 5’ adapters were ligated to each end of the RNA, reverse transcribed, and PCR amplified. The cDNA constructs were fractionated and gel purified. Qubit was used to quantify miRNA and mRNA library. The resulting library (10 nM) was validated with the Agilent 2100 Bioanalyzer. The library was then loaded onto an Illumina MiSeq Flow Cell at a concentration of 8 pM and the samples were sequenced using 50 bp paired-end reads in a High-Seq 2000 Illumina sequencing platform. MiRNA reads were normalized by Reads Per Million (RPM) and mRNA reads were normalized by Fragments Per Kilobase per Million (FPKM). The sequencing quality for miRNA was determined by FastQC.

## RNA isolation and quantitative real-time PCR (qRT-PCR) analysis

Following incubation for 48 h with 10 µM apigenin or 0.1% DMSO, Huh7 cells were harvested for the qRT-PCR experiment. Total RNA was extracted according to the manufacturer’s instructions.The reverse transcription was conducted using a reverse transcription kit (Invitrogen). The expression of hsa-miRNAs and the target genes were measured by qRT-PCR using a SYBR Green PCR Master Mix (Thermo Fisher Scientific Corporation, Foster City, CA, USA) on an ABI-7500 Sequence Detection System (Applied Biosystems, Foster City, CA, USA). snRNA U6 expression in each sample was amplified as hsa-miRNAs internal control, and Glyceraldehyde-3-phosphate dehydrogenase (GAPDH) expression in each sample was amplified as mRNA internal control. Primer 5.0 ViiA 7 Real-time PCR System (Applied Biosystems) software was used to design primers.

# Supplementary Figures and Tables

## Supplementary Figures

**
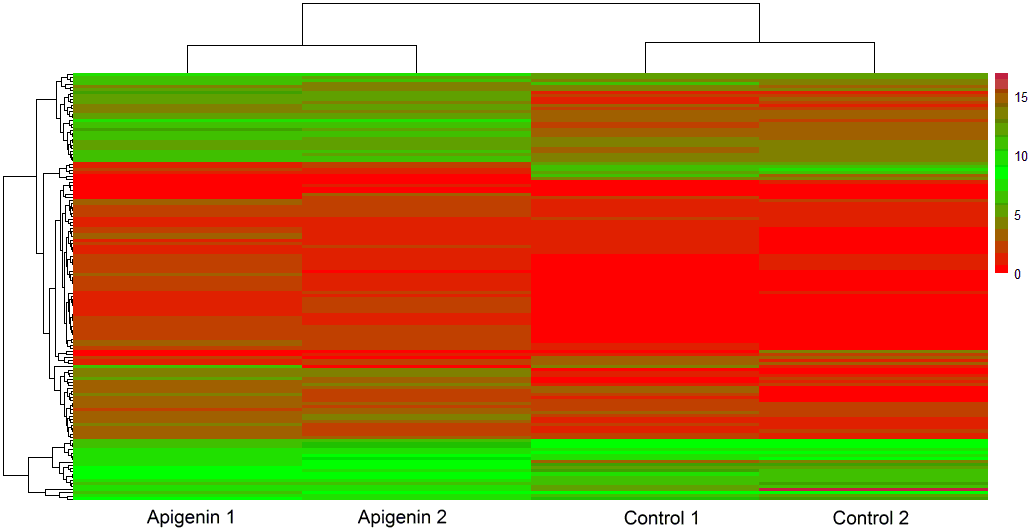
**

**Supplementary Figure 1. Effects of apigenin on the expression profile of miRNAs in Huh 7 cells.** The hierarchical clustering analysis was performed to analyze the expression profile of miRNAs in Huh 7 cells treated with 10 µM apigenin *vs.* 0.1% DMSO. Red indicates high relative expression and green indicates low relative expression.


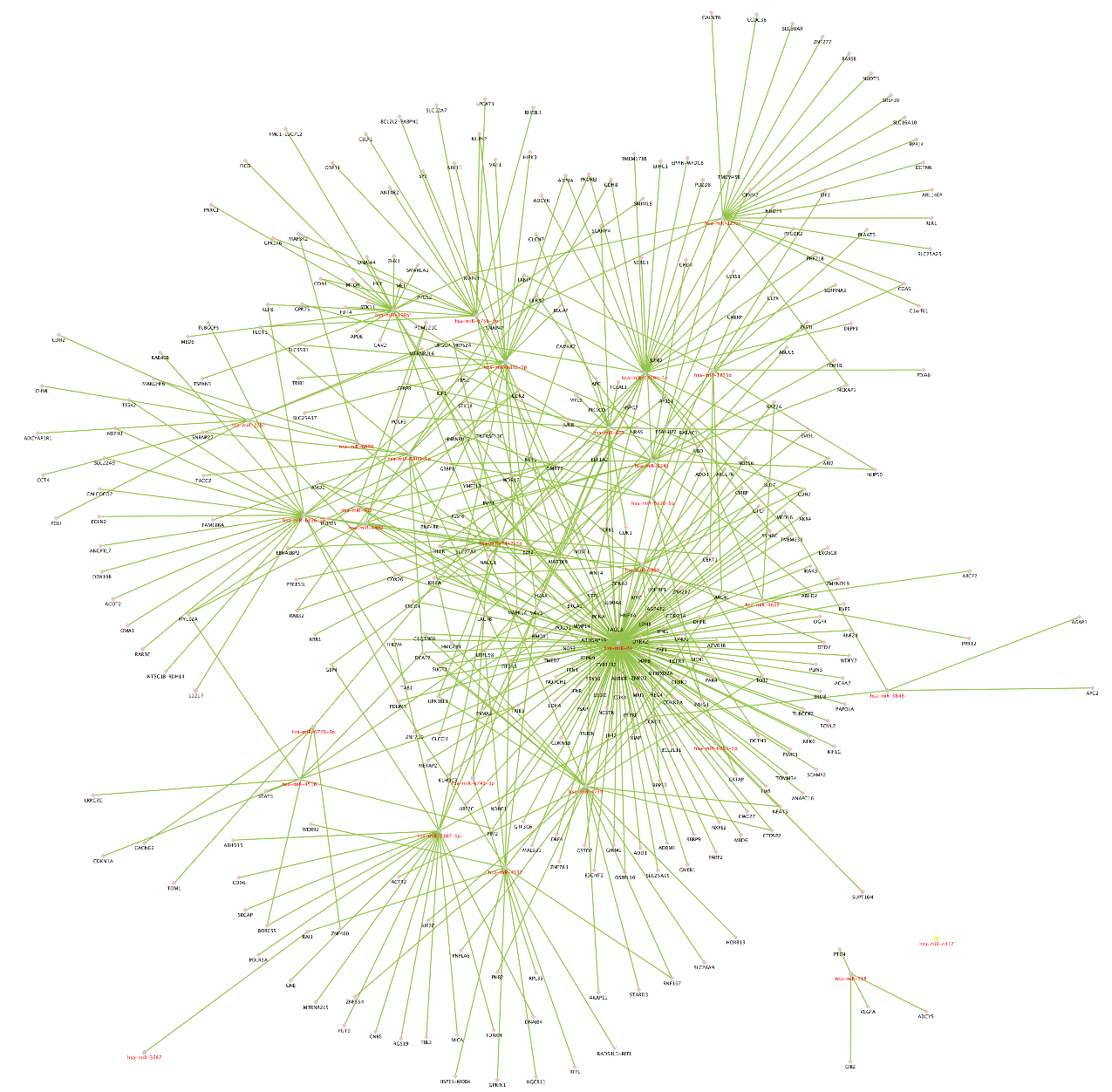


**Supplementary Figure 2. Bioinformatics analysis of differentially expressed miRNAs between apigenin-treated and control groups.** MiRNA-gene networks associated with the up-regulated miRNAs.


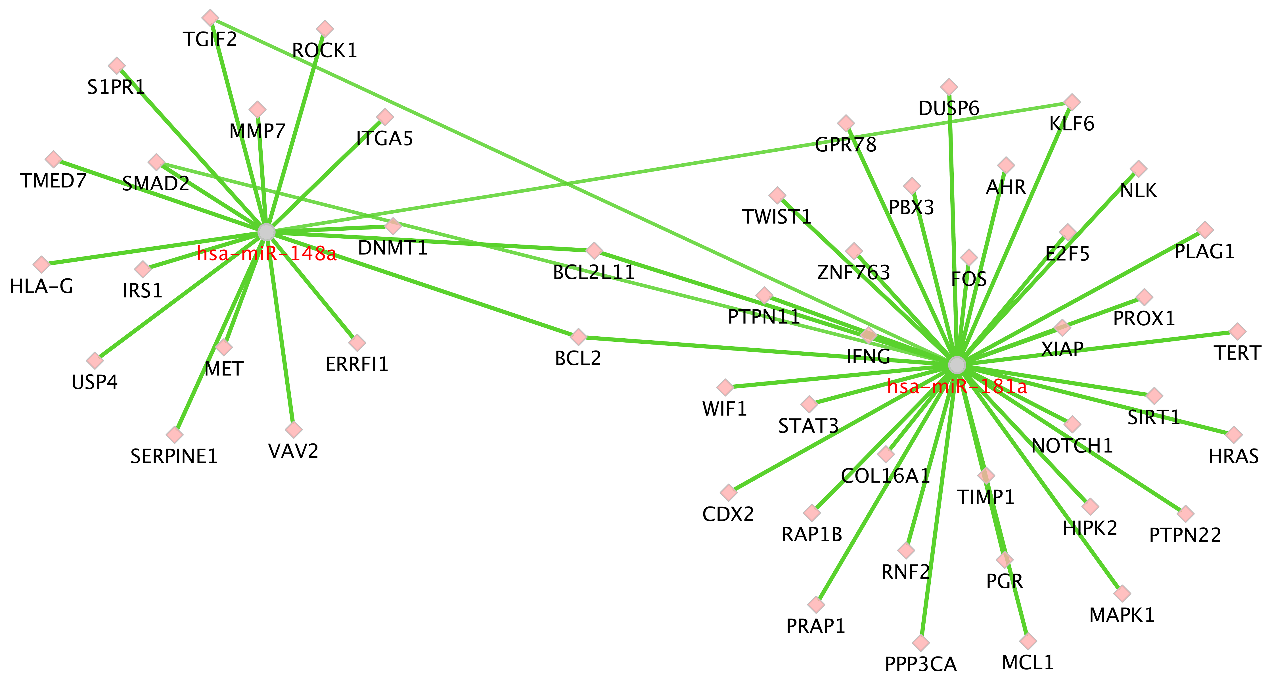


**Supplementary Figure 3. Bioinformatics analysis of differentially expressed miRNAs between apigenin-treated and control groups.** MiRNA-gene networks associated with the down-regulated miRNAs.

## Supplementary Tables

**Supplementary Table 1. Antibodies used in this study.**

| **Antibody** | **Catalog number** | **Dilution** |
| --- | --- | --- |
| Bax | ab81083 | 1:500 |
| Bcl2 | ab194583 | 1:500 |
| anti-Ki67 | ab6526 | 1:1000 |

Note: All antibodies were obtained from Abcam (Cambridge, MA, USA) and Santa Cruz Biotechnology (Santa Cruz, CA, USA).

**Supplementary Table 2. Real-time PCR primer sequences used to test miRNA and target** **genes expression.**

| miRNAs/genes | Primer sequences |
| --- | --- |
| hsa-miR-7847-3p | Forward 5'-GCGTGGAGGACGAGGAGG-3' |
| hsa-miR-663a | Forward 5'-AGGCGGGGCGCCGCGG-3' |
| hsa-miR-1273g-3p | Forward 5'-GGTCCTTCTGCTCCGTCCC-3' |
| hsa-miR-619-5p | Forward 5'-GGGCTGGGATTACAGGCATG-3' |
| hsa-miR-34a-5p | Forward 5'-GGGTGGCAGTGTCTTAGCTGG-3' |
| hsa-miR-5787 | Forward 5'-GGGCTGGGGCGCGGGG-3' |
| hsa-let-7i-5p | Forward 5'-GGGCCTGAGGTAGTAGTTTGTGC-3' |
| hsa-miR-1260b | Forward 5'-GGGATCCCACCACTGCCA-3' |
| hsa-miR-760 | Forward 5'-GGCGGCTCTGGGTCTGTG-3' |
| hsa-miR-215-3p | Forward 5'-GGGTCTGTCATTTCTTTAGGCC-3' |
| hsa-miR-181a-5p | Forward 5'-GAAACATTCAACGCTGTCGGT-3' |
| hsa-miR-148a-3p | Forward 5'-GGGCCTCAGTGCACTACAGAACT-3' |
| MAPK1 | Forward 5'- TGCGCTTCAGACATGAGAACAT-3' |
| PIK3R5 | Forward 5'- CCCTGGTATGAGCGCAATGTA-3' |
| CCND1 | Forward 5'- CACGCGCAGACCTTCGTT-3' |
| ADCY1 | Forward 5'- TACATCCAGAGGCACGACAATG-3' |
| ADCY3 | Forward 5'- GATGAATACGACCACAAGCGTTT-3' |
| GNAQ | Forward 5'- AATGACTTGGACCGCGTAGCT-3' |
| EGF | Forward 5'- CAGGGAAGATGACCACCACTATT-3' |

**Supplementary Table 3. The invasion number of Huh7 cells treated with apigenin.**

| Group | 0.1% DMSO | 5μM Apigenin | 10μM Apigenin | 20μM Apigenin |
| --- | --- | --- | --- | --- |
| Counts 1 | 728 | 650 | 423 | 177 |
| Counts 2 | 743 | 683 | 439 | 203 |
| Counts 3 | 722 | 637 | 415 | 233 |
| average | 731 | 656.6666667 | 425.6666667 | 204.3333333 |
| SD | 10.81665 | 23.71356855 | 12.22020185 | 28.02379941 |
| P(*VS* 0.1% DMSO) |  | 0.00781832 | 0.00000541 | 0.00000700 |

**Supplementary Table 4. Significantly regμlated genes in Huh-7 cells treated with apigenin. (P value ≤ 0.05)**

| ID | Control _ avg _ TPM | Apigenin _avg_ TPM | Log2FoldChange_ apigenin _vs_control | P-value | FDR |
| --- | --- | --- | --- | --- | --- |
| hsa-miR-148a | 32128 | 892.1583603 | -5.17038562 | 1.07E-05 | 0 |
| hsa-miR-181a-5p | 16422 | 535.1124796 | -4.93964447 | 6.36E-05 | 0.01 |
| hsa-miR-1296-3p | 8.0107 | 0.389780121 | -4.36120104 | 1.32E-02 | 0.24 |
| hsa-miR-138-5p | 21.851 | 2.338680727 | -3.22390468 | 1.65E-02 | 0.27 |
| hsa-miR-215-3p | 24.368 | 2.625815566 | -3.21412105 | 2.47E-03 | 0.07 |
| hsa-miR-760 | 26.965 | 3.545476989 | -2.92703092 | 5.37E-03 | 0.13 |
| hsa-miR-4531 | 36.574 | 6.720239957 | -2.44424214 | 2.86E-02 | 0.36 |
| hsa-miR-1247-3p | 16.235 | 3.88220135 | -2.06419465 | 3.72E-02 | 0.44 |
| hsa-miR-1260b | 1078.5 | 267.3161271 | -2.01234266 | 3.01E-02 | 0.38 |
| hsa-let-7i-5p | 6.0953 | 15.23888062 | 1.321984567 | 2.08E-02 | 0.29 |
| hsa-miR-548y | 4.9931 | 12.67651953 | 1.344155743 | 2.63E-02 | 0.34 |
| hsa-miR-4792 | 286.9 | 843.0228799 | 1.555040869 | 3.13E-02 | 0.39 |
| chr19_36513 | 413.22 | 1221.604432 | 1.563781441 | 2.48E-02 | 0.33 |
| hsa-miR-19b-1-5p | 1.6958 | 5.017552053 | 1.565007732 | 4.52E-02 | 0.5 |
| chr16_32766 | 80.037 | 238.2025945 | 1.573442835 | 1.58E-02 | 0.26 |
| hsa-miR-29c-5p | 1.3331 | 4.041368631 | 1.600053685 | 4.31E-02 | 0.49 |
| chr1_783_star | 3.6935 | 11.34107941 | 1.618505834 | 2.24E-03 | 0.07 |
| hsa-miR-1268a | 5.4795 | 16.93290114 | 1.627712043 | 1.55E-02 | 0.26 |
| hsa-miR-6743-5p | 38.428 | 122.6309814 | 1.674107683 | 2.48E-02 | 0.33 |
| hsa-miR-192-3p | 3.3916 | 11.09486671 | 1.709844484 | 1.53E-02 | 0.26 |
| hsa-miR-515-5p | 1.0231 | 3.50802109 | 1.777666705 | 1.17E-02 | 0.21 |
| hsa-miR-4742-3p | 4.0673 | 14.89348888 | 1.872538582 | 6.13E-03 | 0.14 |
| hsa-miR-4634 | 229.91 | 909.7953311 | 1.984492376 | 1.76E-02 | 0.28 |
| chr14_30756 | 48.543 | 192.52576 | 1.987729403 | 1.13E-03 | 0.04 |
| hsa-miR-211-5p | 1.7222 | 6.856874899 | 1.993316884 | 1.21E-02 | 0.22 |
| hsa-miR-5787 | 45.828 | 190.4634928 | 2.055206249 | 1.78E-02 | 0.28 |
| hsa-miR-6775-5p | 1.632 | 6.913396899 | 2.082784964 | 4.88E-03 | 0.12 |
| hsa-miR-5096 | 14.112 | 61.10986378 | 2.114463319 | 1.46E-02 | 0.25 |
| hsa-miR-3907 | 2.5994 | 11.27935627 | 2.117452391 | 4.98E-02 | 0.53 |
| hsa-miR-6087 | 76.807 | 354.8901027 | 2.208071961 | 4.42E-03 | 0.11 |
| hsa-miR-24-3p | 35.733 | 165.5684367 | 2.212108308 | 8.67E-04 | 0.03 |
| hsa-miR-4787-5p | 90.318 | 419.0720806 | 2.214120839 | 1.08E-02 | 0.2 |
| hsa-miR-665 | 2.1101 | 9.802756361 | 2.21587008 | 2.61E-02 | 0.34 |
| hsa-miR-4488 | 30.132 | 144.4625132 | 2.261336669 | 3.21E-02 | 0.39 |
| chr4_11362 | 0.8813 | 4.24145805 | 2.266810817 | 1.39E-02 | 0.24 |
| hsa-miR-7158-5p | 7.8203 | 37.67655451 | 2.268365656 | 4.74E-02 | 0.52 |
| hsa-miR-1469 | 4.6304 | 22.48206583 | 2.279573121 | 2.78E-02 | 0.35 |
| hsa-miR-5095 | 2.0837 | 10.13601448 | 2.282241656 | 1.93E-02 | 0.29 |
| hsa-miR-181a-3p | 139.63 | 686.6154221 | 2.297941215 | 3.52E-03 | 0.09 |
| hsa-miR-34a-5p | 6.1034 | 30.35951253 | 2.314455934 | 4.40E-02 | 0.5 |
| hsa-miR-6895-5p | 1.9419 | 9.746234362 | 2.327347324 | 1.76E-02 | 0.28 |
| chr16_33146 | 8950 | 45729.45307 | 2.353167691 | 1.59E-02 | 0.26 |
| hsa-miR-1273h-5p | 2.6551 | 14.04873925 | 2.403605 | 8.60E-03 | 0.18 |
| hsa-miR-619-5p | 40.993 | 219.5804845 | 2.42130508 | 1.57E-02 | 0.26 |
| hsa-miR-3908 | 9.4929 | 52.60923419 | 2.470391002 | 1.34E-03 | 0.05 |
| hsa-miR-1273e | 1.9947 | 11.11566414 | 2.47837113 | 1.13E-02 | 0.21 |
| chr16_32995 | 564.12 | 3169.310364 | 2.490086828 | 4.20E-03 | 0.11 |
| hsa-miR-1181 | 0.8813 | 5.085531373 | 2.528649049 | 3.42E-02 | 0.42 |
| chr4_9707 | 75.633 | 448.1914835 | 2.567027901 | 2.58E-03 | 0.08 |
| hsa-miR-3656 | 11.371 | 70.36261414 | 2.62946742 | 8.70E-03 | 0.19 |
| hsa-miR-3960 | 311.03 | 1930.194482 | 2.633622192 | 2.02E-03 | 0.06 |
| hsa-miR-4768-3p | 0.9188 | 5.838034433 | 2.667647837 | 4.11E-02 | 0.47 |
| hsa-miR-1343-5p | 8.2904 | 53.34440248 | 2.685831035 | 8.82E-03 | 0.19 |
| hsa-miR-1273g-3p | 59.384 | 382.774296 | 2.688344977 | 7.47E-03 | 0.17 |
| hsa-miR-1972 | 11.917 | 77.56314588 | 2.702370297 | 1.47E-02 | 0.25 |
| hsa-miR-3912-3p | 1.2399 | 8.26376058 | 2.736579453 | 3.49E-02 | 0.42 |
| hsa-miR-4466 | 1.3858 | 9.296466002 | 2.745924101 | 5.86E-03 | 0.14 |
| hsa-miR-6763-3p | 724.13 | 4909.320471 | 2.761195732 | 5.81E-04 | 0.03 |
| hsa-miR-3196 | 26.301 | 181.2748697 | 2.784991309 | 2.75E-03 | 0.08 |
| chr20_36897 | 23.966 | 165.3289365 | 2.786308284 | 1.06E-02 | 0.2 |
| hsa-miR-6789-5p | 0.777 | 5.391732313 | 2.79475506 | 3.73E-02 | 0.44 |
| chr17_34208 | 4.444 | 31.00696267 | 2.802674855 | 2.61E-02 | 0.34 |
| hsa-miR-1237-5p | 1.5276 | 10.66936202 | 2.804096024 | 9.07E-03 | 0.19 |
| hsa-miR-4508 | 13.601 | 96.44773756 | 2.826082778 | 2.95E-03 | 0.08 |
| chr19_36599 | 34559 | 248367.8579 | 2.845355231 | 6.35E-03 | 0.15 |
| hsa-miR-4800-3p | 94.643 | 684.5928766 | 2.854682232 | 6.24E-03 | 0.15 |
| chr9_22988_star | 6.2846 | 46.14598795 | 2.876319327 | 8.36E-03 | 0.18 |
| chr14_31366 | 3.7907 | 28.26357827 | 2.89842002 | 5.43E-03 | 0.13 |
| hsa-miR-199a-3p | 1.3179 | 10.08122739 | 2.935410308 | 5.02E-04 | 0.02 |
| hsa-miR-1538 | 1.0717 | 8.32028258 | 2.956696804 | 2.46E-02 | 0.33 |
| hsa-miR-6836-3p | 259.47 | 2014.821528 | 2.956997047 | 7.61E-04 | 0.03 |
| hsa-miR-370-3p | 0.4407 | 3.601998988 | 3.0310483 | 1.86E-02 | 0.28 |
| hsa-miR-1273a | 5.1238 | 42.70903012 | 3.059262637 | 3.07E-03 | 0.08 |
| hsa-miR-6510-5p | 8.2084 | 71.44943392 | 3.121746481 | 7.43E-04 | 0.03 |
| hsa-miR-7703 | 0.7354 | 6.424437735 | 3.126997898 | 4.27E-02 | 0.49 |
| hsa-miR-6754-3p | 1.009 | 8.8231067 | 3.128330312 | 1.21E-02 | 0.22 |
| hsa-miR-1273g-5p | 0.7506 | 6.564538915 | 3.128505359 | 1.54E-02 | 0.26 |
| hsa-miR-3620-5p | 1.0717 | 9.440033422 | 3.138856245 | 2.12E-02 | 0.29 |
| hsa-miR-1226-5p | 0.5825 | 5.195109133 | 3.156905006 | 1.72E-02 | 0.27 |
| chr17_34206_star | 23.219 | 207.5139592 | 3.159820816 | 1.66E-03 | 0.06 |
| hsa-miR-1281 | 0.4407 | 3.938723349 | 3.159978611 | 2.05E-02 | 0.29 |
| chr19_36635_star | 59.005 | 540.4216408 | 3.195175996 | 1.27E-03 | 0.05 |
| chr5_13840 | 0.5825 | 5.391732313 | 3.210499821 | 1.81E-02 | 0.28 |
| hsa-miR-4492 | 114.84 | 1064.663747 | 3.212741644 | 2.27E-04 | 0.01 |
| hsa-miR-6858-5p | 0.4407 | 4.244924289 | 3.267989346 | 1.02E-02 | 0.2 |
| chr22_38364 | 2.8455 | 27.71116462 | 3.283716238 | 3.03E-03 | 0.08 |
| chr20_36898 | 22.773 | 221.90154 | 3.284554868 | 1.78E-03 | 0.06 |
| hsa-miR-638 | 8.9436 | 88.07439922 | 3.299787737 | 2.86E-03 | 0.08 |
| hsa-miR-4459 | 0.7506 | 7.737345518 | 3.365650377 | 6.95E-03 | 0.16 |
| hsa-miR-4417 | 3.1069 | 32.56502455 | 3.389788012 | 5.13E-04 | 0.02 |
| hsa-miR-149-3p | 0.6088 | 6.534015494 | 3.423849651 | 3.80E-03 | 0.1 |
| hsa-miR-6729-3p | 8.8436 | 98.76735219 | 3.481325599 | 1.71E-04 | 0.01 |
| hsa-miR-4281 | 6.1287 | 68.7140406 | 3.486962221 | 1.40E-03 | 0.05 |
| hsa-miR-1224-5p | 1.3179 | 15.30859128 | 3.53808054 | 4.45E-03 | 0.11 |
| chr7_18754 | 26.182 | 304.2734812 | 3.538724546 | 2.94E-04 | 0.02 |
| hsa-miR-4532 | 6.5582 | 76.87063106 | 3.551059471 | 6.65E-04 | 0.03 |
| hsa-miR-3648 | 4.0146 | 47.48383571 | 3.56412026 | 1.82E-04 | 0.01 |
| hsa-miR-7107-5p | 11.632 | 144.3553431 | 3.633403974 | 2.53E-05 | 0 |
| hsa-miR-4739 | 5.0266 | 63.54493005 | 3.660126976 | 4.66E-05 | 0 |
| hsa-miR-6829-3p | 0.777 | 9.942857542 | 3.677666725 | 1.69E-03 | 0.06 |
| hsa-miR-1343 | 7.5761 | 100.7571749 | 3.733291762 | 3.93E-04 | 0.02 |
| hsa-miR-4516 | 221.39 | 2992.515289 | 3.756724456 | 4.34E-05 | 0 |
| chr5_13734 | 4.0409 | 56.64260817 | 3.809127291 | 5.43E-05 | 0 |
| hsa-miR-3135a | 0.31 | 4.52512665 | 3.867739167 | 2.68E-02 | 0.34 |
| hsa-miR-27b-5p | 2.9105 | 43.19722111 | 3.891607704 | 2.99E-04 | 0.02 |
| hsa-miR-718 | 2.1476 | 32.17976927 | 3.905363878 | 5.22E-04 | 0.02 |
| hsa-miR-3679-5p | 1.0981 | 17.20443612 | 3.969709614 | 1.86E-03 | 0.06 |
| hsa-miR-338-5p | 0.1682 | 2.678871327 | 3.993631367 | 3.15E-02 | 0.39 |
| hsa-miR-1273h-3p | 0.9188 | 14.72218797 | 4.002085309 | 1.00E-03 | 0.04 |
| hsa-miR-6892-3p | 1.632 | 26.48183602 | 4.020321455 | 1.16E-04 | 0.01 |
| hsa-miR-4669 | 0.7243 | 12.40257335 | 4.097965832 | 8.42E-04 | 0.03 |
| hsa-miR-6769b-3p | 0.4407 | 7.737345518 | 4.134089224 | 9.60E-04 | 0.04 |
| hsa-miR-661 | 0.1682 | 3.015595687 | 4.164449094 | 3.44E-02 | 0.42 |
| hsa-miR-663a | 37.771 | 706.3293718 | 4.224995178 | 7.69E-06 | 0 |
| hsa-miR-6756-3p | 1.7333 | 32.96279576 | 4.249251759 | 3.11E-04 | 0.02 |
| hsa-miR-6786-5p | 0.1682 | 3.212218867 | 4.255576276 | 3.63E-02 | 0.43 |
| hsa-miR-567 | 0.1682 | 3.352320048 | 4.317165971 | 3.77E-02 | 0.44 |
| hsa-miR-1273f | 2.341 | 46.97995299 | 4.326858569 | 5.36E-05 | 0 |
| hsa-miR-3131 | 0.31 | 6.227814554 | 4.328506982 | 6.00E-03 | 0.14 |
| chr20_37082 | 1.8001 | 36.28459238 | 4.333180196 | 3.56E-05 | 0 |
| hsa-miR-5689 | 0.1682 | 3.405375808 | 4.3398201 | 1.65E-02 | 0.27 |
| hsa-miR-1273d | 1.3067 | 26.8095107 | 4.358702572 | 4.91E-05 | 0 |
| chr3_7797_star | 0.2836 | 5.978135614 | 4.39772769 | 1.05E-02 | 0.2 |
| hsa-miR-6089 | 4.6163 | 97.93021134 | 4.406955418 | 4.53E-05 | 0 |
| hsa-miR-6090 | 20.608 | 444.4077813 | 4.430640275 | 6.61E-06 | 0 |
| hsa-miR-4783-5p | 0.1682 | 3.658520988 | 4.443266616 | 1.79E-02 | 0.28 |
| hsa-miR-3687 | 31.183 | 696.777353 | 4.481883138 | 1.71E-07 | 0 |
| hsa-miR-490-3p | 0.1418 | 3.212218867 | 4.501602261 | 3.61E-02 | 0.43 |
| hsa-miR-762 | 1.2177 | 29.72005347 | 4.609244581 | 2.37E-05 | 0 |
| hsa-miR-4763-5p | 0.1418 | 3.658520988 | 4.689292601 | 1.78E-02 | 0.28 |
| hsa-miR-378j | 0.1682 | 4.38502547 | 4.704591303 | 1.00E-02 | 0.19 |
| hsa-miR-6753-5p | 0.1418 | 3.798622168 | 4.743508286 | 1.85E-02 | 0.28 |
| hsa-miR-4749-5p | 0.1418 | 3.798622168 | 4.743508286 | 1.85E-02 | 0.28 |
| hsa-miR-6894-5p | 0.1418 | 3.798622168 | 4.743508286 | 1.85E-02 | 0.28 |
| hsa-miR-7847-3p | 6.4235 | 187.4926678 | 4.867322829 | 3.88E-07 | 0 |
| hsa-miR-6743-3p | 4.1675 | 128.7654908 | 4.949425262 | 9.07E-07 | 0 |
| hsa-miR-663b | 0.1682 | 6.144235373 | 5.191239564 | 1.99E-03 | 0.06 |
| hsa-miR-3198 | 0.1682 | 7.150942216 | 5.410139426 | 1.41E-03 | 0.05 |
| hsa-miR-6809-5p | 0.4407 | 21.87313019 | 5.633338313 | 5.30E-06 | 0 |

**Supplementary Table 5. Significantly regμlated genes in Huh-7 cells treated with apigenin. (P value ≤ 0.001)**

| ID | control _avg_TPM | apigenin _avg_TPM | Log2FoldChange_ apigenin _vs_ control | P-value | FDR |
| --- | --- | --- | --- | --- | --- |
| hsa-miR-148a-3p | 32128 | 892.1583603 | -5.17038562 | 1.07E-05 | 0 |
| hsa-miR-181a-5p | 16422 | 535.1124796 | -4.93964447 | 6.36E-05 | 0.01 |
| hsa-miR-24-3p | 35.733 | 165.5684367 | 2.212108308 | 8.67E-04 | 0.03 |
| hsa-miR-6763-3p | 724.13 | 4909.320471 | 2.761195732 | 5.81E-04 | 0.03 |
| hsa-miR-199a-3p | 1.3179 | 10.08122739 | 2.935410308 | 5.02E-04 | 0.02 |
| hsa-miR-6836-3p | 259.47 | 2014.821528 | 2.956997047 | 7.61E-04 | 0.03 |
| hsa-miR-6510-5p | 8.2084 | 71.44943392 | 3.121746481 | 7.43E-04 | 0.03 |
| hsa-miR-4492 | 114.84 | 1064.663747 | 3.212741644 | 2.27E-04 | 0.01 |
| hsa-miR-4417 | 3.1069 | 32.56502455 | 3.389788012 | 5.13E-04 | 0.02 |
| hsa-miR-6729-3p | 8.8436 | 98.76735219 | 3.481325599 | 1.71E-04 | 0.01 |
| hsa-miR-4532 | 6.5582 | 76.87063106 | 3.551059471 | 6.65E-04 | 0.03 |
| hsa-miR-3648 | 4.0146 | 47.48383571 | 3.56412026 | 1.82E-04 | 0.01 |
| hsa-miR-7107-5p | 11.632 | 144.3553431 | 3.633403974 | 2.53E-05 | 0 |
| hsa-miR-4739 | 5.0266 | 63.54493005 | 3.660126976 | 4.66E-05 | 0 |
| hsa-miR-1343 | 7.5761 | 100.7571749 | 3.733291762 | 3.93E-04 | 0.02 |
| hsa-miR-4516 | 221.39 | 2992.515289 | 3.756724456 | 4.34E-05 | 0 |
| hsa-miR-27b-5p | 2.9105 | 43.19722111 | 3.891607704 | 2.99E-04 | 0.02 |
| hsa-miR-718 | 2.1476 | 32.17976927 | 3.905363878 | 5.22E-04 | 0.02 |
| hsa-miR-6892-3p | 1.632 | 26.48183602 | 4.020321455 | 1.16E-04 | 0.01 |
| hsa-miR-4669 | 0.7243 | 12.40257335 | 4.097965832 | 8.42E-04 | 0.03 |
| hsa-miR-6769b-3p | 0.4407 | 7.737345518 | 4.134089224 | 9.60E-04 | 0.04 |
| hsa-miR-663a | 37.771 | 706.3293718 | 4.224995178 | 7.69E-06 | 0 |
| hsa-miR-6756-3p | 1.7333 | 32.96279576 | 4.249251759 | 3.11E-04 | 0.02 |
| hsa-miR-1273f | 2.341 | 46.97995299 | 4.326858569 | 5.36E-05 | 0 |
| hsa-miR-1273d | 1.3067 | 26.8095107 | 4.358702572 | 4.91E-05 | 0 |
| hsa-miR-6089 | 4.6163 | 97.93021134 | 4.406955418 | 4.53E-05 | 0 |
| hsa-miR-6090 | 20.608 | 444.4077813 | 4.430640275 | 6.61E-06 | 0 |
| hsa-miR-3687 | 31.183 | 696.777353 | 4.481883138 | 1.71E-07 | 0 |
| hsa-miR-762 | 1.2177 | 29.72005347 | 4.609244581 | 2.37E-05 | 0 |
| hsa-miR-7847-3p | 6.4235 | 187.4926678 | 4.867322829 | 3.88E-07 | 0 |
| hsa-miR-6743-3p | 4.1675 | 128.7654908 | 4.949425262 | 9.07E-07 | 0 |
| hsa-miR-6809-5p | 0.4407 | 21.87313019 | 5.633338313 | 5.30E-06 | 0 |

**Supplementary Table 6. The most differentially expressed miRNAs in Huh7 cells upon apigenin treatment, as analyzed by Cytoscape-3.7.1 software with ClueGo plug-in and miTarBase databases (Degree value > 16)**

| miRNAs | Degree |
| --- | --- |
| **Up-regulated** |  |
| hsa-miR-24 | 143 |
| hsa-miR-6769b-3p | 26 |
| hsa-miR-6836-3p | 23 |
| hsa-miR-199a-3p | 22 |
| hsa-miR-663 | 22 |
| hsa-miR-4739 | 21 |
| hsa-miR-6892-3p | 20 |
| hsa-miR-7107-5p | 20 |
| hsa-miR-1273g-3p | 19 |
| hsa-miR-1343 | 18 |
| hsa-miR-6089 | 17 |
| **Down-regulated** |  |
| hsa-miR-181a-5p | 25 |
| hsa-miR-148a-3p | 14 |

**Supplementary Table 7. KEGG pathway analysis showing the up-regulated miRNAs-related signaling pathways.(Top 30 pathways)**

| Category | GOTerm | Term PValue |
| --- | --- | --- |
| KEGG_PATHWAY | Hepatocellular carcinoma | 5.82E-10 |
| KEGG_PATHWAY | Pathways in cancer | 7.65E-10 |
| KEGG_PATHWAY | Cellular senescence | 1.68E-09 |
| KEGG_PATHWAY | Bladder cancer | 1.28E-08 |
| KEGG_PATHWAY | Proteoglycans in cancer | 1.77E-08 |
| KEGG_PATHWAY | Gastric cancer | 2.34E-08 |
| KEGG_PATHWAY | Melanoma | 4.30E-08 |
| KEGG_PATHWAY | MicroRNAs in cancer | 6.31E-08 |
| KEGG_PATHWAY | Breast cancer | 1.21E-07 |
| KEGG_PATHWAY | Glioma | 6.16E-07 |
| KEGG_PATHWAY | Chronic myeloid leukemia | 7.07E-07 |
| KEGG_PATHWAY | Pancreatic cancer | 7.07E-07 |
| KEGG_PATHWAY | FoxO signaling pathway | 9.64E-07 |
| KEGG_PATHWAY | Human T-cell leukemia virus 1 infection | 1.34E-06 |
| KEGG_PATHWAY | Human cytomegalovirus infection | 1.99E-06 |
| KEGG_PATHWAY | Colorectal cancer | 2.50E-06 |
| KEGG_PATHWAY | Endometrial cancer | 4.14E-06 |
| KEGG_PATHWAY | Small cell lung cancer | 4.89E-06 |
| KEGG_PATHWAY | Prostate cancer | 8.23E-06 |
| KEGG_PATHWAY | PI3K-Akt signaling pathway | 9.26E-06 |
| KEGG_PATHWAY | AGE-RAGE signaling pathway in diabetic complications | 1.11E-05 |
| KEGG_PATHWAY | Hepatitis B | 1.20E-05 |
| KEGG_PATHWAY | Non-small cell lung cancer | 1.24E-05 |
| KEGG_PATHWAY | Kaposi sarcoma-associated herpesvirus infection | 1.34E-05 |
| KEGG_PATHWAY | Acute myeloid leukemia | 1.40E-05 |
| KEGG_PATHWAY | Epstein-Barr virus infection | 3.37E-05 |
| KEGG_PATHWAY | Viral carcinogenesis | 1.31E-04 |
| KEGG_PATHWAY | Hepatitis C | 1.49E-04 |
| KEGG_PATHWAY | Human papillomavirus infection | 3.28E-04 |
| KEGG_PATHWAY | ErbB signaling pathway | 5.32E-04 |

**Supplementary Table 8. The predicted target genes of the most differentially expressed miRNAs in Huh7 cells upon apigenin treatment based on the results from Cytoscape-3.7.1 software with ClueGo plug-in a**nd KEGG databases.

| Target genes | Degree |
| --- | --- |
| **Up-regulated miRNAs** |  |
| MAPK1 | 43 |
| PIK3CD | 40 |
| HRAS | 37 |
| CCND1 | 37 |
| CDKN1A | 35 |
| E2F2 | 28 |
| MYC | 26 |
| CDK4 | 24 |
| MTOR | 22 |
| CDK2 | 19 |
| STAT3 | 18 |
| PTEN | 18 |
| CDKN1B | 17 |
| IGF1 | 17 |
| MAPK14 | 17 |
| TGFB1 | 17 |
| CDKN2A | 17 |
| VEGFA | 16 |
| WNT4 | 12 |
| CCNA2 | 11 |
| MET | 11 |
| VAV3 | 11 |
| APC2 | 11 |
| APC | 11 |
| **Down-regulated miRNAs** |  |
| MAPK1 | 35 |
| HRAS | 28 |
| STAT3 | 15 |
| FOS | 14 |
| BCL2 | 13 |
| SMAD2 | 12 |
| PPP3CA | 12 |
| IFNG | 11 |
| MET | 9 |
| VAV2 | 8 |

**Supplementary Table 9. KEGG pathway analysis showing the down-regulated miRNAs-related signaling pathways.(Top 30 pathways)**

| Category | GOTerm | Term PValue |
| --- | --- | --- |
| KEGG_PATHWAY | MicroRNAs in cancer | 5.66E-08 |
| KEGG_PATHWAY | Proteoglycans in cancer | 1.38E-06 |
| KEGG_PATHWAY | Cellular senescence | 2.17E-06 |
| KEGG_PATHWAY | FoxO signaling pathway | 6.60E-06 |
| KEGG_PATHWAY | PD-L1 expression and PD-1 checkpoint pathway in cancer | 7.12E-06 |
| KEGG_PATHWAY | Focal adhesion | 1.28E-05 |
| KEGG_PATHWAY | Th17 cell differentiation | 2.36E-05 |
| KEGG_PATHWAY | Human T-cell leukemia virus 1 infection | 2.68E-05 |
| KEGG_PATHWAY | Natural killer cell mediated cytotoxicity | 8.62E-05 |
| KEGG_PATHWAY | Colorectal cancer | 9.63E-05 |
| KEGG_PATHWAY | Apoptosis | 1.03E-04 |
| KEGG_PATHWAY | Gastric cancer | 1.73E-04 |
| KEGG_PATHWAY | AGE-RAGE signaling pathway in diabetic complications | 1.96E-04 |
| KEGG_PATHWAY | T cell receptor signaling pathway | 2.35E-04 |
| KEGG_PATHWAY | Renal cell carcinoma | 4.07E-04 |
| KEGG_PATHWAY | Neurotrophin signaling pathway | 4.36E-04 |
| KEGG_PATHWAY | Th1 and Th2 cell differentiation | 1.38E-03 |
| KEGG_PATHWAY | Chagas disease (American trypanosomiasis) | 2.11E-03 |
| KEGG_PATHWAY | Long-term potentiation | 3.60E-03 |
| KEGG_PATHWAY | Acute myeloid leukemia | 3.60E-03 |
| KEGG_PATHWAY | Yersinia infection | 3.62E-03 |
| KEGG_PATHWAY | Growth hormone synthesis, secretion and action | 3.77E-03 |
| KEGG_PATHWAY | Sphingolipid signaling pathway | 3.77E-03 |
| KEGG_PATHWAY | Prolactin signaling pathway | 3.87E-03 |
| KEGG_PATHWAY | Melanoma | 5.44E-03 |
| KEGG_PATHWAY | Central carbon metabolism in cancer | 1.45E-02 |
| KEGG_PATHWAY | Fc epsilon RI signaling pathway | 1.85E-02 |
| KEGG_PATHWAY | Non-small cell lung cancer | 2.13E-02 |
| KEGG_PATHWAY | VEGF signaling pathway | 2.48E-02 |
